# Kikuchi–Fujimoto Disease in an Old Italian Woman: Case Report and Review of the Literature

**DOI:** 10.1155/2017/7257902

**Published:** 2017-12-14

**Authors:** A. D'Introno, A. Perrone, A. Schilardi, A. Gentile, C. Sabbà, N. Napoli

**Affiliations:** ^1^Clinica Medica “Cesare Frugoni”, Department of Interdisciplinary Medicine, University of Bari “Aldo Moro”, Piazza Giulio Cesare 11, 70124 Bari, Italy; ^2^Institute of Pathological Anatomy, Department of Emergence and Organ Transplantation, University of Bari “Aldo Moro”, Piazza Giulio Cesare 11, 70124 Bari, Italy

## Abstract

Kikuchi–Fujimoto disease (KFD) is a rare, benign, generally self-limiting disease that has higher prevalence in Asian people with a few cases reported in European countries. It generally affects young subjects under 40 years of age and is characterized by regional lymphadenopathy. Here, we present a case of a 66-year-old Italian woman who was extensively examined for right unilateral laterocervical lymph nodes associated with fever, night sweats, fatigue, and weight loss. She was diagnosed as having the KFD only after an excision biopsy of the largest laterocervical lymph node and was then managed symptomatically with NSAIDs. We also made a review of the literature for better awareness of the disease among physicians especially in those countries, like Italy, where the disease is not prevalent and may be frequently misdiagnosed. In fact, to our best knowledge, only seven Italian cases of KFD have been published in the last 15 years with patients being younger than 40 years. We finally highlight that it is noteworthy to consider KFD as differential diagnosis of lymphadenopathy even in old patients, and, since a misdiagnosis of lymphoma is actually feasible, an early biopsy has to be taken into account for confirming diagnosis and helping in the timely and appropriate management.

## 1. Introduction

Kikuchi–Fujimoto disease (KFD) or histiocytic necrotizing lymphadenitis is a rare, benign, generally self-limiting disease that is overexpressed in Asian people, although a worldwide distribution has been reported with a few cases in European countries. The disease usually affects subjects during the third decade of life and frequently manifests as an acute or subacute form with painful posterior cervical lymphadenopathy and systemic symptoms like fever, fatigue, and headache [1]. Its etiology is still unknown, and differential diagnosis includes a spectrum of infectious diseases as well as connective tissue disorders and lymphoproliferative diseases. Here, we describe a case of an old Italian female presented with fever and cervical lymph node enlargement associated with night sweats and weight loss who was finally diagnosed as having KFD by histological examination of the affected lymph nodes.

## 2. Case Presentation

**Figure 1:**
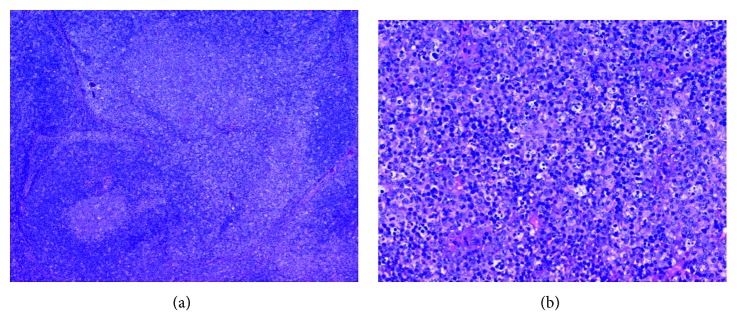
Histopathologic examination showing patchy areas of necrosis, numerous histiocytes, and apoptotic cellular debris. (a) H&E, 20x. (b) H&E, 400x.

**Figure 2:**
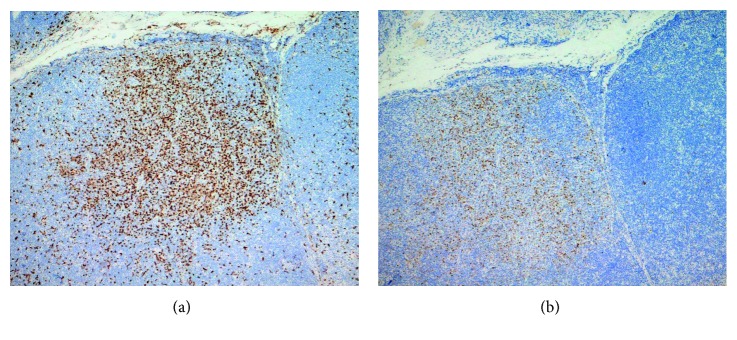
(a) The histiocytic cells are positive for CD68. (b) The histiocytic cells are positive for myeloperoxidase (MPO).

A 66-year-old female patient was admitted to our Internal Medicine Unit on July 2016 with a two-month history of low fever, malaise, fatigue, night sweats, gradual decrease in appetite, and body weight loss of 4 kilograms. She also complained of painful right laterocervical lymph nodes for 1 month. Her clinical history was unremarkable except for a hypothyroidism diagnosed in 2013 and treated with a daily dose of levothyroxine. She had no past history of tuberculosis or contact with it. On admission, her heart rate was 90 bpm, blood pressure was 110/60 mmHg, respiratory rate was 16 breaths/minute, and body temperature was 37.5°C. Physical examination revealed two right nontender, fixed, and painful laterocervical lymph nodes. There were no hepatosplenomegaly or other clinically appreciable lymphadenopathy elsewhere. Examination of respiratory and other systems was normal. Laboratory analysis showed slight increase in C-reactive protein (CRP), lactate dehydrogenase (LDH), and erythrocyte sedimentation rate (ESR) values that were, respectively, of 20 mg/L (n.v. < 2.9), 705 UI/L (n.v. < 450), and 54 mm/h. Thyroid hormones and neoplastic markers were all within the normal range. Antinuclear antibody, anti ds-DNA, and rheumatoid factor were negative; HIV, cytomegalovirus IgM, herpes virus IgM, Epstein–Barr virus IgM, Brucella, and Toxoplasma were all negative. Quantiferon exam was also negative. Chest X-ray was unremarkable. Ultrasonography revealed enlarged right-sided hypoechoic laterocervical (maximum size of 1.7 cm) and submental lymph nodes (size 1 cm) with low peripheral flow, eccentric cortical thickening, and deviation of the hila. A total body CT scan was then performed to better assess lymphadenopathy, and it confirmed the results of the ultrasounds. Because of a dilemma in clinical diagnosis and the persistence of symptoms, an excision biopsy of the largest laterocervical lymph node was performed. Histological examination showed paracortical necrotic foci with the presence of small to large CD3+, CD5+/−, CD4−/+, CD8+/− T lymphocytes, in addition to numerous histiocytes expressing CD68PGM1/MPO and abundant apoptotic nuclear debris. No polymorphonuclear neutrophils and no caseous necrosis were observed (Figures 1 and 2). On the basis of these morphological and immunophenotype findings, a diagnosis of Kikuchi–Fujimoto disease was made.

The patient was treated symptomatically with NSAIDs, and at two-month follow-up, malaise, fatigue, and appetite were improved; she denied fever and night sweats, and ultrasonography showed a decrease in the size of laterocervical lymph nodes, whereas submental lymph nodes were not detected. ESR, CRP, and LDH were also restored at normal range. At six-month follow-up, the patient was free of symptoms, and laterocervical lymph nodes were no more detectable on ultrasonography.

## 3. Discussion

KFD is a benign disease more prevalent in Asian populations that affects predominantly young adults. While a worldwide distribution and an involvement of all ages have been reported [1], a few cases have been described in European countries generally involving young subjects. Particularly, to our best knowledge, only seven Italian cases of KFD have been published in the last 15 years with all patients being younger than 40 years [2–8].

Patients with KFD commonly present with nonspecific signs and symptoms mainly characterized by enlargement of unilateral cervical lymph nodes and fever. Less common symptoms are weakness, night sweats, anorexia, weight loss, arthralgia, or cutaneous manifestations [1, 9]. An involvement of other lymph nodes has been described, and extranodal manifestations affecting skin, eye, bone marrow, or liver as well as disseminated disease with fatal outcome have been reported in a very few cases [10–13].

The etiopathogenesis of KFD is still unknown. An autoimmune contribution to the pathogenesis has been suggested, and lymphocyte-infecting viruses have been postulated to have a causative role, even though there is not convincing evidence so far. Bacteria and parasites have also been implicated in the pathogenesis with controversial results [14, 15].

There are not specific laboratory analyses or instrumental examination that can suggest a diagnosis of KFD. Leukopenia with sometimes atypical peripheral blood lymphocytes, anemia, or an increase in inflammatory pattern index has been usually observed [16, 17]. An increase in LDH and alanine aminotransferase has been sometimes documented.

Imaging findings are usually nonspecific and can mimic not only lymphoma but also various nodal diseases with necrosis. Ultrasounds, CT scan, and magnetic resonance in any case showed particular signs with a distinctive lymphadenopathy pattern consisting of many small clustered lymph nodes [9, 18, 19]; however, these observations must be interpreted cautiously. Fine-needle aspiration cytology (FNAC) has been performed in some cases, but it has limited diagnostic potential with an overall diagnostic accuracy for KFD estimated at about 56% [20–22]. Therefore, biopsy is today the only way to have a correct diagnosis of KFD.

In this report, we described a case of an old adult female with typical cervical lymphadenopathy accompanied by fever who also complained of fatigue, night sweats, and body weight loss. No variations in laboratory analyses were observed, except of slight ESR, CRP, and LDH elevation. Due to the age and the clinical history and examination of the patient, the first diagnostic hypothesis was a lymphoma although the enlarged lymph nodes in lymphoma are usually painless.

A differential diagnosis including viral and bacterial diseases as well as systemic lupus erythematosus was also considered, but microbiological analysis and autoimmunity examination were all negative.

The ultrasound and CT scan evidenced only right-sided laterocervical and submental limph node enlargement. To rule out the suspected diagnosis of lymphoproliferative disease, and before starting any specific therapy, we decided to perform a biopsy of the largest laterocervical lymph node.

Unexpectedly, the histological examination excluded a diagnosis of lymphoproliferative disease and showed typical features of KFD, that is, necrotic paracortical foci formed by different cellular types, predominantly histiocytes and small or large lymphocytes with absence of neutrophils and granulomatous reaction at the margin of the necrotic areas. The histopathological diagnosis was also confirmed by the immunophenotype of KFD that typically consists of a predominance of T cells and relatively few B cells and NK cells and histiocytes expressing histiocyte-associated antigens, myeloperoxidase, and CD68.

It is of note that one year before the hospital admission, the patient was diagnosed to have an autoimmune thyroiditis with hypothyroidism, thus supporting a possible autoimmunity pathogenesis of the KFD and a relationship between these two pathologies, although only a few KFD cases with autoimmune thyroiditis have been reported [3, 23, 24].

Usually, the pathology is self-limiting with no therapy administration; however, reports suggest the use of NSAIDs or corticosteroids in case of persistent or more severe symptoms. Therapy with colchicine has also been reported in some cases [1].

In our case, we decided to start therapy with NSAIDs after the histological results because of persistence of fever, fatigue, and night sweats. After one week of therapy, the patient denied fever, and malaise and night sweats were improved. At six-month follow-up, the patient was free of all symptoms.

In conclusion, an original feature of this case report was the diagnosis of KFD in an elderly Italian woman, being the pathology actually rare in Italy and especially in old subjects. Then, our report suggests that it is noteworthy to consider KFD as differential diagnosis of lymphadenopathy even in old patients and in countries where the disease is not prevalent. Since a misdiagnosis of lymphoma is actually feasible, we highlight that if one patient presents with lymph node enlargement eventually associated with other nonspecific signs and symptoms, an early biopsy has to be taken into account for confirming diagnosis and helping in the timely and appropriate management.

Finally, we speculate that KFD may be underdiagnosed in clinical practice mainly in European countries because it is rare and underrecognized; in fact, mild forms of low-grade fever and typical laterocervical lymph node enlargement with spontaneous resolution could be wrongly diagnosed as infection disease. Hence, the recognition of KFD is important to avoid misdiagnosis.
